# 遗传性球形红细胞增多症骨髓红系造血代偿特征

**DOI:** 10.3760/cma.j.issn.0253-2727.2022.02.005

**Published:** 2022-02

**Authors:** 小霞 李, 园 李, 馨 赵, 广新 彭, 建平 李, 蕾 叶, 文睿 杨, 康 周, 慧慧 樊, 洋 杨, 佑祯 熊, 洋 李, 琳 宋, 丽萍 井, 莉 张, 凤奎 张

**Affiliations:** 中国医学科学院血液病医院（中国医学科学院血液学研究所），实验血液学国家重点实验室，国家血液系统疾病临床医学研究中心，天津 300020 State Key Laboratory of Experimental Hematology, National Clinical Research Center for Blood Diseases, Institute of Hematology & Blood Diseases Hospital, Chinese Academy of Medical Sciences & Peking Union Medical College, Tianjin 300020, China

**Keywords:** 球形红细胞增多, 遗传性, 骨髓红系造血代偿, 网状细胞计数, Spherocytosis, hereditary, Bone marrow compensatory erythropoiesis, Reticulocyte count

## Abstract

**目的:**

揭示遗传性球形红细胞增多症（HS）骨髓红系造血的代偿特征，探究不同程度贫血对骨髓造血代偿的影响。

**方法:**

收集2014年7月至2020年9月中国医学科学院血液病医院确诊的HS患者临床及实验室资料，以外周血网织红细胞绝对值作为替代参数，评估骨髓红系造血代偿能力，并对不同贫血程度HS患者红系造血代偿进行比较。

**结果:**

① 302例HS患者中代偿性溶血病（代偿组）115例，轻、中、重度贫血（失代偿组）患者分别为74、90、23例。② 失代偿组血清红细胞生成素（EPO）水平与HGB呈负相关（*rs*＝−0.585，*P*<0.001）。③ HS患者的中位网织红细胞计数（ARC）0.34（0.27，0.44）×10^12^/L，约为正常人的4.25倍，最大ARC 0.81×10^12^/L，约为正常人的10倍；代偿组的中位ARC 0.29（0.22，0.38）×10^12^/L，约为正常人的3.63倍，失代偿组中位ARC 0.38（0.30，0.46）×10^12^/L，明显高于代偿组（*z*＝4.999，*P*＝0.003），达正常人的4.75倍。④代偿组的ARC与HGB呈负相关（*r*＝−0.177，*P*＝0.002）；失代偿组ARC与HGB呈正相关（*rs*＝0.191，*P*＝0.009），轻、中、重度贫血组间ARC差异无统计学意义（*χ*^2^＝4.588，*P*＝0.101）。⑤ 轻、中、重度贫血组的中位未成熟网织红细胞指数（IRF）分别为13.1％（9.1％，18.4％）、17.0％（13.4％，20.8％）、17.8％（14.6％，21.8％），轻度贫血组IRF小于中度及重度贫血组（*P*_adj_值均<0.05），而中度和重度贫血组间差异无统计学意义（*P*_adj_＝1.000）；轻、中、重度贫血组的中位新生网织红细胞计数分别为5.09（2.60，7.74）×10^10^/L、6.24（4.34，8.83）×10^10^/L、7.00（3.07，8.22）×10^10^/L，组间差异无统计学意义（*χ*^2^＝3.081，*P*＝0.214）。

**结论:**

HS骨髓红系造血代偿随不同程度红细胞减少而增加，维持HGB在正常水平；而一经贫血发生，造血代偿即已达最大水平，不因贫血加重、EPO增加而进一步代偿增加。

## Abstract (en)

**Objective:**

To reveal the compensatory features of bone marrow（BM）erythropoiesis in hereditary spherocytosis（HS）and to explore the effect of diferent hemoglobin levels on this compensation.

**Methods:**

Clinical and laboratory data of patients with HS were collected, and the peripheral blood absolute reticulocytes counts value was taken as the surrogate parameter to evaluate the ability of erythropoiesis compensation. BM erythropoiesis compensation in HS with diferent degrees of anemia were evaluated.

**Results:**

①Three hundred and two patients were enrolled, including 115 with compensated hemolytic disease, 74 with mild anemia, 90 with moderate anemia, and 23 with severe anemia. ②Hemoglobin（HGB）was negatively correlated with serum erythropoietin in the decompensated hemolytic anemia group（EPO; *rs*＝−0.585, *P*<0.001）. ③The median absolute reticulocyte count（ARC）of HS patients was 0.34（0.27, 0.44）×10^12^/L, up to 4.25 times that of normal people. The maximum ARC was 0.81×10^12^/L, about 10 times that of normal people. The median ARC of patients with compensated hemolytic disease was 0.29（0.22, 0.38）×10^12^/L, up to 3.63 times that of normal people. The median ARC of patients with hemolytic anemia was 0.38（0.30, 0.46）×10^12^/L, which was significantly higher than the patients with compensated hemolytic disease, up to 4.75 times that of normal people（*z*＝4.999, *P*＝0.003）. ④ ARC was negatively correlated with HGB in the compensated hemolytic disease group（*rs*＝−0.177, *P*＝0.002）and positively correlated with HGB in the decompensated hemolytic anemia group（*rs*＝0.191, *P*＝0.009）. There was no significant difference in the ARC among patients with mild, moderate, and severe anemia（*χ*^2^＝4.588, *P*＝0.101）. ⑤The median immature reticulocyte production index of the mild, moderate, and severe anemia groups was 13.1％（9.1％, 18.4％）, 17.0％（13.4％, 20.8％）, and 17.8％（14.6％, 21.8％）, respectively; the mild anemia group had lower index values than the moderate and severe anemia groups（*P*_adj_ values were both<0.05）, but there was no significant difference between the latter groups（*P*_adj_＝1.000）. The median immature reticulocyte count of patients in the mild, moderate, and severe groups was 5.09（2.60, 7.74）×10^10^/L, 6.24（4.34, 8.83）×10^10^/L, and 7.00（3.07, 8.22）×10^10^/L, respectively; there was no significant difference among the groups（*χ*^2^＝3.081, *P*＝0.214）.

**Conclusion:**

HGB can be maintained at a normal level through bone marrow erythropoiesis, while red blood cells are reduced in HS. However, once anemia develops, the bone marrow exerts its maximum erythropoiesis capacity and does not increase, regardless of anemia aggravation or serum EPO increase.

贫血或溶血致使组织氧张力下降，红细胞生成素（EPO）增加，进而促使骨髓红系造血代偿增加，并诱导未成熟的网织红细胞提前从骨髓释放，外周血网织红细胞计数（ARC）显著增加[1]–[3]。ARC反映骨髓红系有效造血，其最大增加程度可大致反映机体红系的造血代偿能力。关于贫血严重程度与骨髓红系造血代偿关系的报道[4]–[6]非常少，且年代久远。遗传性球形红细胞增多症（HS）是由于红细胞膜骨架蛋白缺陷引起的遗传性溶血性疾病，稳定状态下表现为代偿性溶血病或不同严重程度的溶血性贫血；大多数HS患者在儿童和青年时期即被诊断，较少合并其他异常[7]–[10]。HS病理生理机制主要包括：①红细胞膜内在缺陷；②脾脏选择性阻留、损伤和清除缺陷的红细胞。在骨髓幼红细胞阶段，细胞核和其他细胞骨架蛋白可稳定细胞膜，HS溶血主要是脾脏破坏缺陷的成熟红细胞所致[8],[11]。本研究中我们假定HS患者骨髓具有正常的造血代偿功能，同时除外其他已知可能影响骨髓造血的因素，以此作为探究贫血严重程度与骨髓红系代偿关系的模型，探讨骨髓红系造血代偿特征。

## 病例与方法

1. 病例资料：收集2014年7月至2020年9月我院门诊及住院明确诊断为HS患者的临床及实验室检查资料，HS诊断参照英国HS诊治指南（2011年版）[9]。凡具有以下任意一项者不纳入研究：①造血原料（铁、血清叶酸、血清维生素B_12_）缺乏；②接受过EPO、G-CSF治疗；③1个月内输注过红细胞；④心、肝、肾等重要脏器功能异常；⑤近1个月内合并感染；⑥脾脏切除。HS贫血严重程度及脾脏大小参照文献[12]定义，代偿性溶血病：HGB≥120 g/L（男性）、HGB≥110 g/L（女性）；轻度贫血：100 g/L～<120 g/L（男性）、HGB 100 g/L～<110 g/L（女性）；中度贫血：HGB 80 g/L<100 g/L；重度贫血：HGB<80 g/L。

2. 外周血细胞参数及其他血清学指标检测：ARC、未成熟网织红细胞指数（IRF）、HGB检测由全自动模块式血液体液分析仪（日本希森美康公司）完成，ARC正常参考值为（0.024～0.084）×10^12^/L；血清EPO、铁蛋白、间接胆红素、血清叶酸、血清维生素B_12_检测使用CouterDX1800全自动化学发光免疫分析仪（美国Beckman公司）完成，血清EPO参考值为2.59～18.50 U/L。IRF正常参考值5％～22％。新生的网织红细胞计数（IRC）＝IRF×ARC。

3. 统计学处理：应用SPSS 26.0软件进行统计学分析，符合正态分布的计量资料以均值±标准差表示，组间比较采用方差分析；非正态分布的计量资料以中位数（*P*25，*P*75）表示，组间比较采用Kruskal-Wallis *H*检验或Mann-Whitney *U*检验，多组间两两比较采用Bonferroni法进行校正。无序分类变量采用卡方检验或Fisher精确概率法进行差异性分析，有序分类变量采用Kruskal-Wallis *H*检验进行差异性分析。连续变量的相关性分析采用Pearson相关性分析（变量符合正态分布）及 Spearman 秩相关（变量不满足正态分布）。双侧*P*<0.05为差异有统计学意义。

## 结果

1. HS患者的一般特征：共302例HS患者纳入分析，其中男144例（47.7％），女158例（52.3％），中位年龄24（13，35）岁。患者中位HGB 107（92，123）g/L，其中代偿性溶血病组（代偿组）115例，中位HGB 128（120，138）g/L；失代偿贫血组（失代偿组）187例，中位HGB 95（85，106）g/L。轻度贫血74例，中位HGB 107（104，110）g/L；中度贫血90例，中位HGB 91（85，95）g/L，重度贫血23例，中位HGB 72（64，78）g/L。90例患者进行了脾脏超声检查，84例（93.3％）脾脏肿大，其中轻度肿大14例（15.5％）、中度肿大42例（46.7％）、重度肿大28例（31.1％）。

2. HS患者血清EPO水平：104例HS患者检测了血清EPO水平，中位血清EPO水平为67.59（37.71，119.98）U/L，代偿组中位血清EPO水平为41.52（28.54，74.02）U/L，明显低于失代偿组的97.83（46.93，142.78）U/L（*z*＝3.824，*P*<0.001），轻、中、重度贫血患者中位血清EPO水平分别为52.13（34.96，75.91）、120.08（95.47，155.95）、268.71（97.83，283.35）U/L，组间差异有统计学意义（*χ*^2^＝17.083，*P*<0.001）。相关性分析显示104例HS患者血清EPO与HGB水平呈负相关（*rs*＝−0.560，*P*<0.001），其中代偿组EPO与HGB无明显相关性（*rs*＝−0.263，*P*＝0.106），而失代偿组EPO与HGB呈负相关（*rs*＝−0.585，*P*<0.001）。

3. HGB水平与新生红细胞：HS患者中位ARC 0.34（0.27，0.44）×10^12^/L，约为正常人的4.25倍；最大ARC为 0.81×10^12^/L，约为正常人的10倍。代偿组的中位ARC 0.29（0.22，0.38）×10^12^/L，约为正常人的3.63倍，而失代偿组中位ARC为0.38（0.30，0.46）×10^12^/L，为正常人的4.75倍，明显高于代偿组（*z*＝4.999，*P*＝0.003）。轻、中、重度贫血组中位ARC分别为0.40（0.32，0.49）×10^12^/L，0.37（0.30，0.46）×10^12^/L，0.37（0.28，0.40）×10^12^/L，组间差异无统计学意义（*χ*^2^＝4.588，*P*＝0.101）。

302例HS患者的ARC变异系数（CV_ARC_）为36％，其中代偿组CV_ARC_为41％，失代偿组CV_ARC_为32％。相关性分析显示全部HS患者ARC与HGB呈负相关（*r*＝−0.177，*P*＝0.002），其中代偿组ARC与HGB呈负相关（*r*＝−0.186，*P*＝0.047），而失代偿组ARC与HGB呈正相关（*r*＝0.191，*P*＝0.009）。

全部HS患者中位IRF为13.3％（9.2％，18.3％），其中代偿组中位IRF为10.3％（7.7％，13.4％），明显低于失代偿组的16.0％（10.8％，20.2％）（*z*＝6.827，*P*<0.001）；轻、中、重度贫血组患者中位IRF分别为13.1％（9.1％，18.4％）、17.0％（13.4％，20.8％）、17.8％（14.6％，21.8％），组间差异有统计学意义（*z*＝0.274，*P*<0.001），其中轻度贫血患者中位IRF小于中度及重度贫血组（*P*_adj_值均<0.05），而中度和重度贫血组患者中位IRF组间差异无统计学意义（*P*_adj_＝1.000）。相关性分析显示全部HS患者IRF与HGB呈负相关（*r*s＝−0.448，*P*<0.001）；其中代偿组ARC与HGB呈负相关（*r*＝−0.215，*P*＝0.021），失代偿组IRF与HGB呈负相关（*r*s＝−0.260，*P*<0.001）。

HS患者中位IRC 4.61（2.55，7.27）×10^10^/L；代偿组患者的中位IRC 2.94（1.65，4.79）×10^10^/L，小于失代偿组患者的中位IRC 5.92（3.33，8.37）×10^10^/L（*z*＝−7.002，*P*<0.001）；轻、中、重度贫血组患者中位IRC分别为5.09（2.60，7.74）×10^10^/L、6.24（4.34，8.83）×10^10^/L、7.00（3.07，8.22）×10^10^/L，组间差异无统计学意义（*χ*^2^＝3.081，*P*＝0.214）；相关性分析显示全部HS患者IRC与HGB呈负相关（*r*s＝−0.400，*P*<0.001），代偿组IRC与HGB呈负相关（*r*s＝−0.263，*P*＝0.005），失代偿组IRC与HGB无明显相关性（*r*s＝−0.090，*P*＝0.222）。

4. 骨髓红系造血代偿能力不同的HS贫血患者临床特征比较：以中位ARC 0.38×10^12^/L为界值将HS失代偿贫血患者分为两组，分析影响红系代偿能力的相关因素。结果见表1，性别、年龄、血清EPO、脾脏肿大组间差异均无统计学意义（*P*值均>0.05），而贫血严重程度组间明显不同，ARC>0.38×10^12^/L的患者HGB水平更高（*P*＝0.036）。

**表1:** 不同红系造血代偿能力的HS失代偿贫血组患者临床及实验室特征比较

组别	例数	男/女	年龄 [岁，*M*（*Q*1，*Q*3）]	EPO [U/L，*M*（*Q*1，*Q*3）]	HGB [g/L，*M*（*Q*1，*Q*3）]	IBIL [U/L，*M*（*Q*1，*Q*3）]	脾脏肿大^a^[例（％）]			
							无	轻度	中度	重度
ARC≤0.38×10^12^/L组	93	33/60	24（8，35）	96.65（38.68，136.95）	93（84，102）	68.3（42.9，108.3）	1（1.1）	3（3.2）	13（14.0）	6（6.5）
ARC>0.38×10^12^/L组	94	41/53	18.5（8，30）	100.31（54.08，146.03）	98（88，107）	74.2（52.7，105.8）	1（1.1）	4（4.3）	13（13.8）	12（12.8）

*P*值		0.323	0.302	0.477	0.036	0.236	0.740			

注：HS：遗传性球形红细胞增多症；ARC：网织红细胞计数；IBIL：间接胆红素。a：53例患者进行了脾脏超声检测

## 讨论

我们对肾功能正常、造血原料充足的HS患者骨髓红系造血的代偿特征进行了研究，结果表明：①骨髓具有强大的红系造血代偿能力，中位代偿能力达正常人的4.25倍，最大可达10倍；②骨髓红系造血代偿能力个体间差异较大；③ 代偿性溶血病HS患者ARC与HGB呈负相关，提示骨髓红系造血尚未达到最大代偿；④失代偿贫血HS患者ARC不因贫血加重、血清EPO水平升高而进一步增加，表明一旦贫血即已达骨髓红系最大造血代偿；⑤红系造血代偿能力强大的HS患者贫血相对较轻。

骨髓红系造血代偿需依靠肾脏分泌EPO刺激[13]–[15]，本研究中代偿性溶血病组患者血清EPO明显升高，失代偿组患者血清EPO水平明显高于代偿组，且血清EPO水平与HGB呈负相关，贫血程度越重相关性越强，提示在骨髓红系造血代偿过程中，EPO代偿性生成适当增加，血清EPO水平并非限制骨髓红系造血代偿的因素。

正常人骨髓红系每日新生约2×10^11^个红细胞（占红细胞总数1％）以补充外周血中因衰老或凋亡而被清除的红细胞，在贫血和溶血时骨髓加速生成红细胞，慢性溶血性贫血患者骨髓红细胞生成可达正常情况的4～7倍，最大可达正常情况的8～10倍[4]–[6],[16]–[17]。本研究结果显示HS患者骨髓红系造血中位代偿能力达正常人的4.25倍，最大可达10倍，与上述文献报道一致，证实骨髓具有强大的红系造血代偿能力。另外，我们计算出造血代偿及失代偿HS患者骨髓红系造血具体的代偿倍数，为评估骨髓红系是否达到其最大代偿能力提供参考。更重要的是，我们发现代偿组ARC与HGB水平呈负相关，且代偿组ARC小于失代偿组，提示轻度溶血时骨髓红系未发挥其最大造血代偿即可维持HGB在正常范围；失代偿组血清EPO水平随贫血加重明显上升，但轻、中、重度贫血组的ARC组间并无差异，提示贫血一旦发生，骨髓红系造血代偿即已达最大，不因贫血加重、血清EPO水平升高而代偿增加。除此之外，我们还发现HS患者骨髓红系造血代偿能力个体间差异较大，在失代偿组中，贫血程度与骨髓红系造血代偿能力呈正相关，ARC更高的患者贫血相对较轻，即骨髓造血代偿能力是影响患者表现型的重要原因。

IRF代表新生的更为年轻的网织红细胞构成比，在造血衰竭、骨髓抑制、造血原料缺乏经有效治疗后及骨髓移植后比ARC能更早地反映骨髓红系造血恢复，是目前常用的监测骨髓造血恢复的敏感指标[18]。在我们的研究中，几乎所有患者IRF都在正常范围内，有研究发现是由于膜蛋白缺失干扰球形细胞通透性导致荧光染料不能充分进入缺陷的网织红细胞，使未成熟的网状细胞被错误地分类为更成熟的部分，导致IRF假性减少[19]，提示HS患者的实际IRF高于检测值。既往研究显示外周血红细胞减少后，正常骨髓红系代偿性增生，幼稚的网织红细胞提前从骨髓释放，网织红细胞在外周血中成熟时间延长，且贫血越重成熟时间越长[20]，即外周血新生的网织红细胞越多。本研究结果显示轻度贫血组IRF小于中度贫血组，但中、重度贫血组IRF差异无统计学意义，提示贫血后，网织红细胞在一定程度上提早释放到外周血。IRC即新生的网织红细胞绝对数，在轻、中、重度贫血组并无差异，提示一旦出现贫血骨髓即达最大代偿，新生的网织红细胞并不因贫血加重而生成增加。Hillman等[20]认为网织红细胞生成指数反映贫血患者骨髓红系代偿能力较正常人的代偿倍数，由于它的提出是基于网织红细胞成熟时间随贫血加重延长的理论，而我们通过分析IRF及IRC与贫血程度的关系发现，贫血后骨髓在一定程度上释放新生网织红细胞的速率增加，释放的总量并未增加，所以我们认为网织红细胞生成指数不能很好反映贫血患者骨髓红系代偿能力。

迄今，贫血程度与骨髓红系造血代偿的关系尚未被完全阐明，我们以HS患者为研究对象证实，轻度红细胞减少时，可经骨髓代偿维持HGB在正常水平；而一旦发生贫血，骨髓红系造血代偿能力即已达最大，不再受贫血严重程度、血清EPO水平的影响。
